# Genome-Wide Identification and Systematic Analysis of the *HSF* Gene Family in *Capparis spinosa* and Its Expression Under High Temperature

**DOI:** 10.3390/ijms27010497

**Published:** 2026-01-03

**Authors:** Li Li, Ruiqi Zhang, Aybulan Tuohtarbek, Cong Cheng

**Affiliations:** 1 Xinjiang Key Laboratory for Ecological Adaptation and Evolution of Extreme Environment Organisms, College of Life Sciences, Xinjiang Agricultural University, Urumqi 830052, China; lili@xjau.edu.cn; 2 College of Life Science, Xinjiang Agricultural University, Urumqi 830052, China

**Keywords:** *HSF* gene family, *Capparis spinosa*, heat stress, expression, gene structure, systematic evolution

## Abstract

The *heat shock transcription factor* is a critical transcription factor gene family in plant response to biotic and abiotic stress, especially in regulating high-temperature stress. While this gene family has been extensively characterized and investigated across a broad range of plant species, research focusing on desert plants with extreme stress tolerance remains relatively scarce. Therefore, this study aimed at the desert plant *Capparis spinosa*, conducted the whole genome identification of its *HSF* gene family, and performed a comprehensive systematic analysis including gene structure, chromosome localization, systematic evolution, gene collinearity, and other characteristics. The results showed that the *CsHSF* family contains 24 genes that are distributed on 14 chromosomes. It has three types, as usual, and different types of genes contain specific conserved motifs. The *CsHSF* genes exhibit concentrated collinearity with *Arabidopsis thaliana*, and upstream of the genes, there are 605 *cis*-elements in response to growth and development, stress, and hormones. On this basis, heatmaps and co-expression networks were drawn based on the reported gene expression in different growth regions of the *Capparis spinosa* genome. The results demonstrated that certain genes exhibit distinct expression patterns across different growth regions and have close interrelationships with each other. Further transcriptome sequencing and analysis were performed on the leaves of wild *Capparis spinosa* exposed to high-temperature stress, and the exploration of differential expression of the *CsHSF* genes revealed that 8 genes play significant regulatory roles in response to heat stress. The results of this research can provide valuable insights into the function and mechanism of the *HSF* gene family in desert plants, as well as a reference for the analysis of stress resistance mechanisms in desert plants. The obtained genes can supply candidate genes for subsequent functional verification and mechanism analysis.

## 1. Introduction

Hot extremes are one of the climate disasters that can lead to the deterioration of plant habitats and large-scale crop yields, thereby affecting ecological stability and agricultural development [1]. The intensity and frequency of extreme high temperatures have increased steadily in recent years, becoming one of the primary factors that compromise global food production stability [2]. *Heat shock transcription factor* (*HSF*) is a key factor in the signal transduction system of eukaryotes involved in responding to heat stress and various abiotic stresses [3]. The genes of this family not only enhance plant stress tolerance through regulating the transcriptional activity of downstream functional genes, but also have been shown to play an indispensable role in plant growth and development [4].

The HSF gene family has been identified in no fewer than 111 plant species, and dedicated database platforms for the analysis and investigation of this family have also been reported [5]. Research shows that the average number of genes in this family of plants is 26.58, but there are significant differences among species. Only 13 *HSF* genes were identified in *Amaranthus hypochondriacus* [6], while they range from 61 to 82 in wheat [3,7,8,9,10]. The conserved core structure of this gene family is characterized by a DNA-binding domain (DBD) localized at the N-terminus, which enables specific recognition and binding to the heat shock element (HSE) with the consensus sequence 5′-nGAAnnTTCn-3′ within the promoter region of target genes. The Oligomerization domain (OD) immediately following is responsible for HSF protein trimerization and protein–protein interactions during transcriptional activation. In addition, the nuclear localization signal (NLS) and nuclear export signal (NES) at the C-terminus of the gene are responsible for regulating the shuttle process of *HSF* genes between the nucleus and cytoplasm [3]. Based on the specific conserved domains and motifs distribution, this gene family can be classified into three major categories (A, B, and C), which can be further subdivided into 16 distinct groups (A1-9, B1-5, C1-C2). Among these, the A-type has the aromatic and hydrophobic amino acid residues (AHAs) activator motifs at the C-terminal region, and the B-type possesses the tetrapeptide LFGV motif as the repressor domain (RD) [4]. The gene functions differed greatly between each type due to structural differences [3,11]. The A1-type HSF protein is usually retained in the cytoplasm in an inactivated form complexed with heat shock protein (HSP), including HSP70 and HSP90. Under stress conditions, misfolded proteins separate HSP from the complex, and the released HSF protein is transported to the nucleus to form an active trimer for related gene transcription [12]. The A2- and A3-type HSFs amplify this effect downstream of the A1-type HSF protein. However, the B-type HSF genes may have a dual regulatory function, including reducing plant basal heat tolerance and enhancing acquired heat tolerance [13,14,15].

The number of *HSF* genes varies greatly among different species, with only one *HSF* gene found in the yeast and the Drosophila, and only four in vertebrates. But in plants, there are usually dozens or even hundreds of *HSF* genes. For example, 621 *HSF* genes were identified from 13 cotton genomes [16], and 88 *HSF* genes of sunflower [17]. The larger number of *HSF* genes in plants is related to their sessile lifestyle, which enhances plant resistance to multiple stresses. Meanwhile, the variable number of *HSF* genes in different plants also reflects the highly differentiated function of plant *HSF* genes. Therefore, identifying the *HSF* gene family across different plant species holds considerable significance for a comprehensive understanding of its function. However, current research on the *HSF* gene family is focused on model plants and conventional crops. In the established plant *HSF* database (http://hsfdb.bio2db.com (accessed on 14 November 2025)), the 111 species reported are mostly horticultural plants and economic crops [5]. In desert plants with strong stress resistance, relatively few studies have been conducted on the *HSF* gene family; with the exception of *Ammopiptanthus mongolicus*, where 24 *HSF* genes were identified and analyzed [18]. Therefore, extensive analysis of the characteristics of the *HSF* gene family in desert plants, focusing on its differences from conventional crops, can provide new insights into the study of plant stress tolerance function. Therefore, the *HSF* gene family in plants with strong stress resistance needs more research effort.

*Capparis spinosa* Linn., also known as the caper bush, is widely distributed in the Mediterranean Sea and Xinjiang, Gansu, and Xizang provinces in China [19]. As a typical desert plant, the caper can tolerate extreme environments, including high temperatures, drought, poor fertility, and wind erosion [20]. Its main root can grow vertically downwards to a maximum depth of 30–40 m, and its branches are clustered and can grow horizontally or diagonally up to 2–3 m. Because of its strong ability to gather sandy soil, the caper becomes a pioneer plant in combating desertification [21]. Meanwhile, the pickled flower buds of caper can be consumed as seasonings, its leaves can be used as high-nutrient feed, and its roots, stems, and leaves can be used as medicine to treat diseases such as rheumatism [22]. Therefore, this species has significant value for research and application.

Extensive studies have been conducted on the capers by researchers, encompassing physiological responses to drought and salt stress [23,24], as well as thermotolerance characteristics [25]. In 2022, the first high-quality reference genome sequencing of *Capparis spinosa* at the chromosome level was completed, which not only revealed the evolutionary characteristics but also identified several heat shock protein genes, providing references and laying the foundation for further in-depth analysis of its stress resistance mechanism [26]. However, the *HSF* gene family of *Capparis spinosa* has not been genome-wide identified and reported.

In the present study, the *Capparis spinosa HSF* gene family is genome-wide identified and comprehensively studied through analysis of gene structure, systematic evolution, and expression patterns. Furthermore, key functional genes are identified through expression analysis under high-temperature conditions. The research results obtained can not only lay a theoretical foundation for the in-depth study of *HSF* gene function in *Capparis spinosa* but also provide information for the mechanism analysis of extreme high-temperature adaptability, and can also provide genetic resources for the improvement of high-temperature-tolerant varieties in other crops.

## 2. Results

### 2.1. The Identification of the CsHSF Gene Family

Totally 24 putative *HSF* genes were identified from the *Capparis spinosa* genome via nucleotide Blast and protein HMMER search methods, and were renamed as *CsHSF01*-*CsHSF24*. The specific information, including gene length, number of amino acids, molecular weight, theoretical isoelectric point (pI), and subcellular localization of these genes, is presented in Table 1, and the renamed information is shown in Table A1. Among all identified *CsHSF* genes, the coding sequence length ranged from 399 to 1557 bp, with the number of exons varying from 2 to 4. The corresponding CsHSF proteins spanned 132 to 518 amino acids in length, and the pI fell within the range from 4.98 to 9.22, with the molecular weights ranging from 42.69896 kDa to 57.32964 kDa. The subcellular localization prediction result indicated that all CsHSF proteins were targeted to the nucleus, except for the *CsHSF04* gene, which was predicted to be localized in both the nucleus and the chloroplast simultaneously.

**Figure 1:**
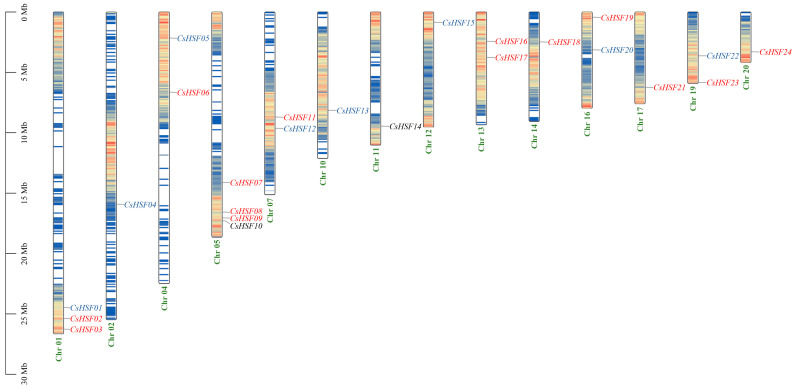
The chromosome location of *CsHSF* genes. The type of *CsHSF* genes was shown with different font colors, with red representing type A, blue representing type B, and black representing type C.

On the genome of *Capparis spinosa*, these 24 *CsHSF* genes were localized on 14 chromosomes, and all genes tended to be located in gene-dense regions (Figure 1). Especially, the B-type genes, *CsHSF01*, were gathered with the A-type genes, *CsHSF02* and *CsHSF03,* on Chr 01. And the C-type genes of *CsHSF10* were gathered with the A-type genes, *CsHSF08* and *CsHSF09*, on Chr 05.

### 2.2. The Structure of the CsHSF Gene Family

**Figure 2:**
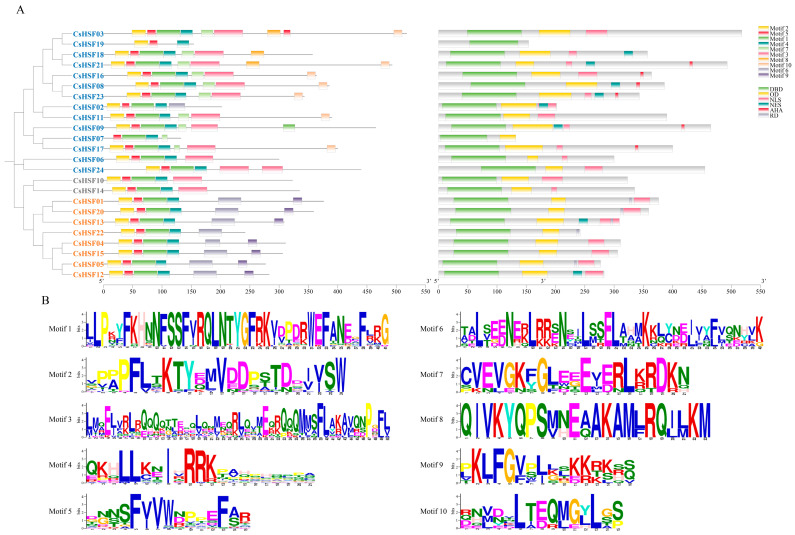
The structure of the CsHSF protein. (**A**) The clustering was conducted by MEGA, and 3 different types were shown with font colors, with blue representing type A, orange representing type B, and gray representing type C. The conserved domains on the left were identified by Conserve Domain Search of NCBI, and the conserved motifs on the right were identified by MEME. DBD, DNA-binding domain; OD, Oligomerization domain; NLS, Nuclear localization signal; NES, Nuclear export signal; AHA, Aromatic and hydrophobic amino acid residues; and RD, Repressor domain. (**B**) The conserved motifs of CsHSF proteins identified by MEME.

Integrating the conserved domain and motif features, the *CsHSF* gene family was subdivided into three types as usual, among which 14 genes belong to the A type, 7 belong to the B type, and 2 belong to the C type. The sequence structure of the *CsHSF* family is shown in Figure 2. The *CsHSF* genes of the same type display a similar structure composition and location, implying the structural basis for the functional differences between different types. Through the MEME analysis, ten conserved motifs were identified. The distribution showed that they were mostly contained in the conserved domains. But some of them, such as motif 7, which is extremely conservative, were not set at the location of the conserved domain.

### 2.3. The Collinearity Analysis of the CsHSF Gene Family

**Figure 3:**
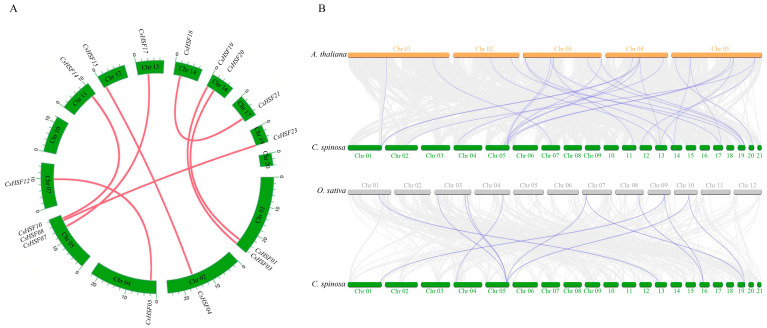
The collinearity analysis of *CsHSF* genes within *Capparis spinosa* (**A**), and the collinearity of *CsHSF* with *AtHSF*, as well as with *OsHSF* (**B**).

Eight pairs of homologous genes within the *HSF* family of *Capparis spinosa* were found through collinearity analysis (Figure 3A), suggesting that the family may have undergone whole-genome duplication events during evolution, thereby expanding the family and simultaneously facilitating its function. The pairwise synteny analysis with *Arabidopsis thaliana* (Arabidopsis) and *Oryza sativa* (rice) revealed that 17 *CsHSF* genes exhibited collinearity with 19 *AtHSF* genes, and 6 *CsHSF* genes were collinear with 10 *OsHSF* genes (Figure 3B). Among them, the *CsHSF* gene on Chr 05 chromosome exhibits concentrated collinearity with Arabidopsis and rice, indicating that the *HSF* gene at this position is relatively conserved in evolution among different species, suggesting its function across multiple plants.

### 2.4. Systematic Evolution Analysis of the CsHSF Genes

**Figure 4:**
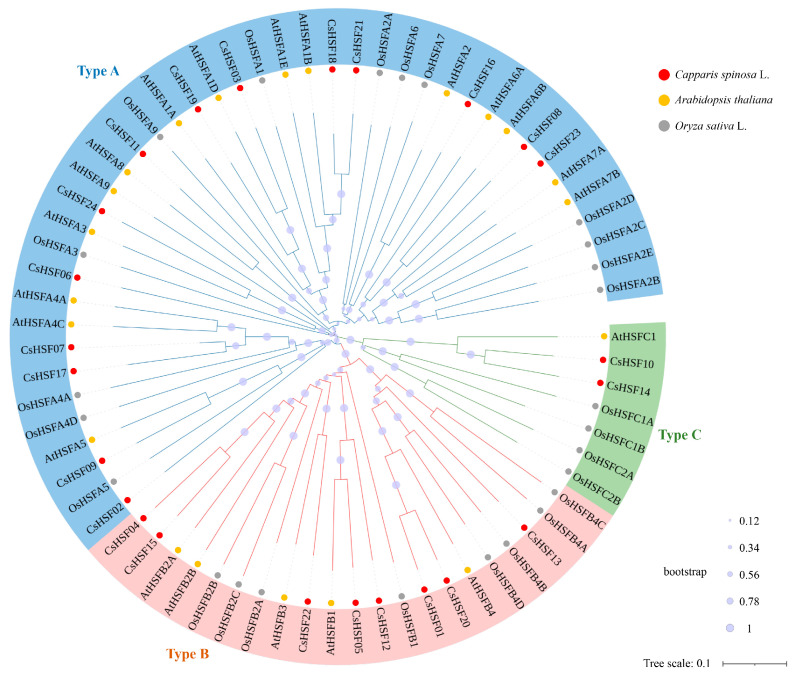
The Systematic evolution of *HSF* genes in multiple plant species. The background color of the genes represents each gene type, and the color of the dots in front of the genes represents different species. The dots on the branch showed the bootstrap value as the figure caption indicated.

Multiple sequence alignment was conducted on the *CsHSF* gene family using Arabidopsis and rice as outgroup species, and a phylogenetic tree was plotted. Out-group species genes in the phylogenetic tree are provided in Table A2, with the results shown in Figure 4. The bootstrap value indicates that the evolutionary tree has high credibility. The background color of the genes represents the *HSF* gene type, and the color of the dots in front of the genes represents different species. From this evolutionary tree, *HSF* genes of the same type are located on the same branch in multiple species, indicating that the formation of each type of *HSF* in this family occurred earlier than the differentiation time of the three species mentioned above. The branch lengths of the evolutionary tree are relatively uniform, indicating that there is little difference in the type of gene differentiation within this family. Among them, Class A *HSF* has 4 branches, and Class B *HSF* has 2 branches, indicating that different types of HSF may form more structurally and functionally conserved subclasses.

To further evaluate the purification pressure faced by this gene family during evolution, the Ka/Ks value was predicted, with the results summarized in Table 2. Eight gene pairs obtained from collinearity analysis were used to calculate the Ka/Ks ratio, and seven of them were successfully predicted. The Ka/Ks values were less than 1 (ranging from 0.0289 to 0.3321) in all gene pairs, implying that the purifying selection may have occurred during the *CsHSF* family evolution.

### 2.5. The Cis-Regulatory Elements of the CsHSF Gene Promoter

**Figure 5:**
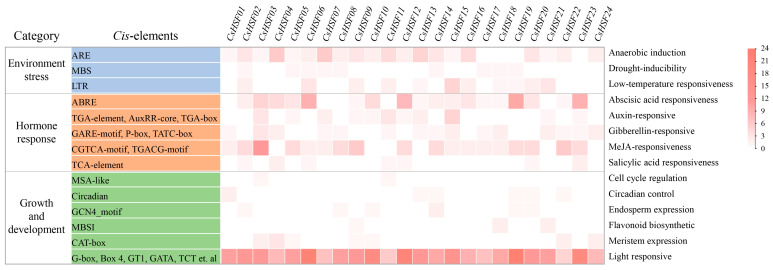
The statistics of *cis*-regulatory elements upstream of the *CsHSF* genes.

The *cis*-regulatory elements of the *CsHSF* gene promoter sequence were systematically identified using the PlantCARE website. The results are shown in Figure 5. A total of 605 *cis*-elements were found in the *CsHSF* gene promoter. Specifically, 87 elements responded to environmental stress, including heat, drought, cold, and oxidation. The number of hormone-responsive elements is 191. And there are up to 327 growth and development response elements. Notably, the *CsHSF* gene promoter region contains abundant photoresponsive elements. Further analysis revealed that the *CsHSF03* gene has more jasmonic acid methyl ester response elements, while the *CsHSF06*, *CsHSF12*, *CsHSF19*, and *CsHSF23* genes have more abscisic acid response *cis*-elements.

### 2.6. Protein Interaction Network of the CsHSF Genes

**Figure 6:**
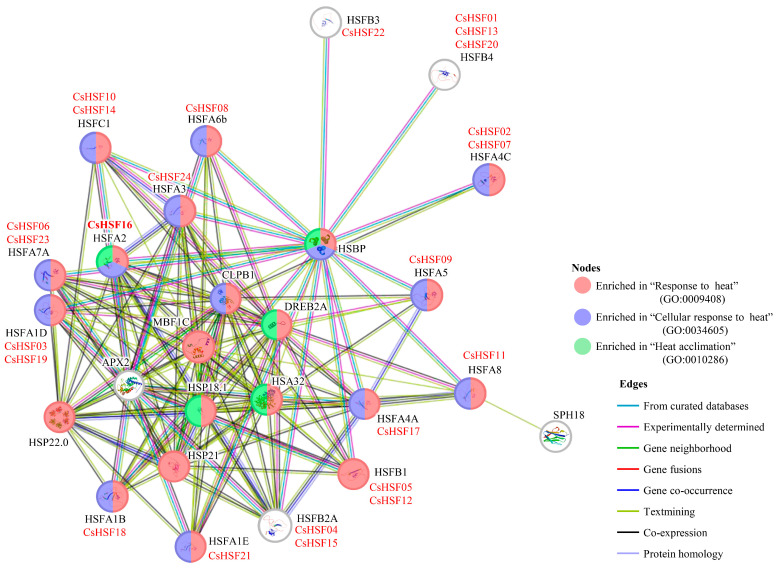
The protein interaction network elements of *CsHSF*.

The HSF protein interaction network was constructed based on the STRING database, and the results are shown in Figure 6. All HSF protein nodes in the network are enriched to varying degrees in heat stress-related functions, including “Response to heat”, “Cellular response to heat”, and “Heat accumulation”. The *CsHSF16* gene was simultaneously annotated in three heat stress-related functional directions, while multiple other *HSF* genes were annotated in two functional directions.

### 2.7. The Heatmap and Co-Expression Network of CsHSF Genes

**Figure 7:**
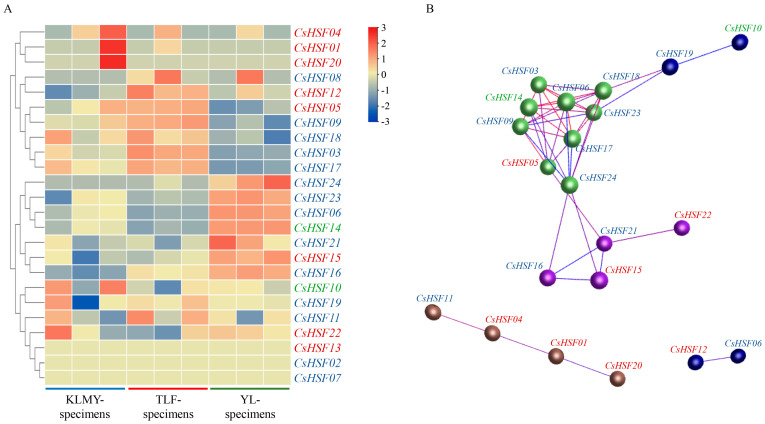
The heatmap (**A**) and co-expression network (**B**) of *CsHSF* genes at multiple growing regions. The *CsHSF* genes were shown in font colors for types, with the blue representing type A, dark red representing type B, and green representing type C. The nodes’ color represents the gene cluster. The co-expression network was constructed with *p*-value ≤ 5.0 × 10^−2^.

Based on the *CsHSF* gene expression in the *Capparis spinosa* genome database, a heatmap and co-expression network were constructed, as shown in Figure 7A. The gene expression was based on specimens collected from Ili, Turpan, and Karamay (hereafter referred to as YL-specimens, TLF-specimens, and KLMY-specimens throughout the text, tables, and figures). Some *CsHSF* genes exhibit completely different expression patterns in samples with distinct regions. For example, the gene cluster including *CsHSF08*, *CsHSF12*, *CsHSF05*, *CsHSF09*, *CsHSF18*, *CsHSF03*, and *CsHSF17* was upregulated in TLF-specimens and downregulated in YL-specimens. On the contrary, the gene cluster of *CsHSF24*, *CsHSF23*, *CsHSF06*, *CsHSF14*, *CsHSF21*, *CsHSF15*, and *CsHSF16* was upregulated in YL-specimens and downregulated in TLF-specimens. It can be inferred that the expression of genes in this family is involved in environmental responses of the regions, especially in the process of stress resistance and other functions. Meanwhile, both gene clusters contain multiple types of *HSF* genes, indicating a wide range of interactions between *CsHSF* genes. Therefore, a significant co-expression network was constructed, as shown in Figure 7B. The network is composed of three sub-networks, and the division of genes in sub-networks is basically consistent with the clustering in the heatmap. This result demonstrated that the genes in the three sub-networks may have closer interactions with each other.

### 2.8. The Expression Analysis of CsHSF Genes Under High-Temperature

**Figure 8:**
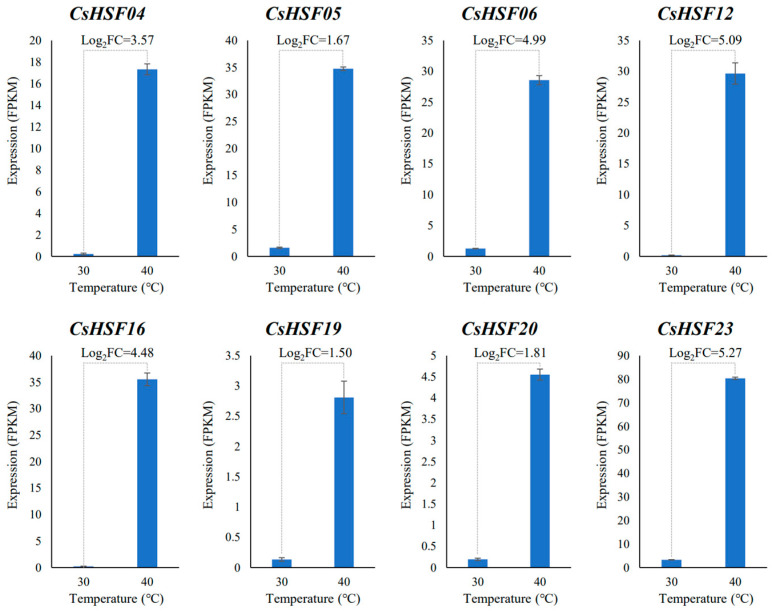
The FPKM expression and the fold change in differentially expressed *CsHSF* genes at 40 °C high temperature.

To further identify the core genes of *CsHSF* responsive to heat, the leaves of wild *Capparis spinosa* plants were subjected to transcriptome sequencing at high temperature (40 °C). Among the differential expression gene analysis results, 8 *CsHSF* genes were found to be significantly differentially expressed under high temperature. The gene expression and fold change in these 8 genes were shown in Figure 8. These 8 *CsHSF* genes were all upregulated DEGs, indicating that the increased expression in *CsHSF* genes can improve the high-temperature adaptive capacity of *Capparis spinosa*. The differential expression fold of these 8 genes ranged from 1.5 to 5.27, among which *CsHSF23* expression was significantly upregulated from 3.3 to 80.36 as the temperature increased from 30 °C to 40 °C. Therefore, *CsHSF23* is likely to play a crucial role in the high-temperature stress response of *Capparis spinosa*, and this functional role requires further experimental verification.

## 3. Discussion

This study focuses on the stress-tolerant desert plant, *Capparis spinosa*, and identifies 24 *CsHSF* genes from this species. In the gene identification, we used multiple methods, including nucleotide Blast and HMMER alignment, to ensure all genes were comprehensively screened out from the genome. The combination of the two methods has been reported in the gene family identification of growth-regulating factors in *Dendrobium officinale* and *Dendrobium chrysotoxum* [27] and in the *CqFAR1* gene in quinoa [28], which has been proven to have high accuracy. The verification of conserved domains after identification using the NCBI website improved the accuracy of identification. As a result, 24 *CsHSF* genes were obtained from the *Capparis spinosa* genome. This result is consistent with the number of most plants, such as 21 in Arabidopsis [29], 25 *HSF* genes in rice [30], 25 in *Verbena bonariensis* [31], and 24 in *Ammopiptanthus mongolicus* [18]. This result suggests that the strong stress resistance of *Capparis spinosa*, particularly its high-temperature tolerance, may not depend on the extensive replication and amplification of the *HSF* gene. And previous reports have shown that the losses of *HSF* genes are more than duplication in higher plants after the whole-genome duplication [5]. Meanwhile, the lack of redundancy in the number of genes also leads to the compound function of *HSF* genes in *Capparis spinosa*. These findings may lay the groundwork for subsequent in-depth investigations into the evolution and regulatory mechanisms of this gene family.

Previous studies have reported the functional diversity between different types of *HSF* genes, including the activation cycle model of A1-type *HSF* genes acting with HSP70/90 in regulating plant heat tolerance [12], the amplification effect of A2- and A3-type HSFs which downstream the A1-type genes [32,33], as well as the B-type HSFs in reducing basal heat tolerance but enhancing the ability to acquire heat tolerance [13,15,34]. In this study, we obtained 14 A-type, 7 B-type, and 2 C-type *CsHSF* genes. The number of each gene type is consistent with the results in other plants, especially the C-type genes, which have only two in most plants [30]. In the chromosome localization analysis, we found the phenomenon of multiple *CsHSF* genes of different types clustering in adjacent positions on chromosomes. From an evolutionary standpoint, the clustering of *HSF* genes on chromosomes may originate from duplication events of ancestral genes, which then adapt to different stress responses through subfunctionality or neofunctionalization [35]. In terms of function, due to the distinct functions of different *HSF* types, this clustered distribution may facilitate coordinated regulation; for instance, via shared cis-elements, the formation of regulatory networks or coordinated regulation of chromatin accessibility [5].

In the conserved motif analysis, the *CsHSF* gene family was found to have several unique conserved structures different from those of other plant species. Existing studies have performed motif analyses on 94 *HSF* genes from 7 representative plants, spanning from lower to higher plants [5]. The results showed that 10 common motifs were found in the family, but the number of shared motifs decreased as the evolutionary complexity increased. From moss plant *P. patens* with ten motifs, to lower plant *C. reinhardtii* with only six motifs (M1–M6), to Arabidopsis with only one motif (M3), and rice with only two motifs (M1 and M2) [5]. By comparison, among the ten conserved motifs identified for *CsHSF* in this study, five motifs (M1, M2, M3, M5, M10) are fully consistent with the previously reported motifs, and three motifs (M4, M7, M9) exhibit partial overlap with the reported motifs, and M6 and M8 are completely distinct from the those identified in the seven aforementioned species. From this perspective, the *Capparis spinosa HSF* gene structure is more primitive and shares greater sequence similarity with those in lower plants. And the unique conserved motifs, M6 and M8, may be associated with the environmental adaptability of *Capparis spinosa* to arid habitats.

From the perspective of systematic evolution, previous studies reported that the plant *HSF* family expansion mainly relies on whole-genome replication (WGD) and the segmental duplication events. For example, the cotton *HSF* family expands through dispersal, fragmentation, tandem, and proximal replication events, and the polyploidization process generates cotton-specific orthologous gene clusters. After the replication event, the *HSF* gene family underwent strong purification selection during evolution, retaining its conserved function [36]. And the *HSF* genes of different species exhibit independent branching evolution on the phylogenetic tree, indicating that each branching gene evolves independently after species differentiation [5]. The results obtained here on the *HSF* gene family of *Capparis spinosa* are consistent with the above. The evolutionary tree of multiple species shows that each types of *HSF* genes are distributed on different branches, and within the same type, *HSF* genes of specific species tend to separate from each other, indicating the independent evolution of *HSF* gene families among plant species. In the collinearity analysis, we found four pairs of A-type, three pairs of B-type, and one pair of C-type homologous *CsHSF*, which implied that all C-type and most of the B-type *CsHSF* have undergone whole-genome duplication events during evolution. In contrast, A-type genes may not have undergone such a high probability of replication, indicating that, in the process of evolution, the functions of B-type and C-type may have been more urgently needed and varied, while A-type functions have somehow met the survival needs of *Capparis spinosa*. In collinearity analysis, three *CsHSF* genes on Chr 05 and seven genes in Arabidopsis exhibit relatively dense collinearity. Among these seven Arabidopsis genes, *AT3G63350.1* has been validated to respond to salt stress by binding to the E-box element [37], and *AT5G45710.1* has the function of responding to heat stress by alternative splicing [38]. This can provide a reference for the function of *CsHSF* genes in the genome of *Capparis spinosa*.

To investigate the environmental and upstream factors that affect the expression level of the *CsHSF* gene, we analyzed the *cis*-acting elements in the gene promoter region, in which 87 elements that responded to environmental stress, including heat, drought, cold, and oxidation, were found. However, there was no enrichment of HSEs upstream of the *CsHSF* gene. Although some other species possess HSEs in their own upstream regions as binding sites for HSF proteins, the presence of HSEs in the promoters of their downstream target genes is of greater functional importance. The lack of enrichment of HSEs in the *CsHSF* promoter in this study only indicates that the mutual regulatory interaction within this family is relatively weak and does not impair the ability of this gene family to respond to heat stress. In addition, some special species (such as *R. tomentosa*) may evolve atypical regulatory modes, with their *HSF* promoter containing “stress-related *cis*-elements” instead of the typical HSE [39]. In the future, we will also conduct more in-depth research on whether this new *cis*-element exists in *Capparis spinosa*.

To further explore the functional roles of the *CsHSF* family, we analyzed its expression based on the genomic association expression data and the transcriptome of *Capparis spinosa* leaves collected from different temperature environments of the wild. Comparing the three regions of KLMY, TLF, and YL, TLF is the hottest with a maximum temperature of over 47 °C, followed by KLMY with the extremely high temperatures of up to 40 °C, while YL is relatively mild in temperature and rarely experiences high temperatures above 35 °C. Previous reports have shown that different *HSF* members have tissue-specific expression patterns and exhibit differential responses to different stresses [5]. However, this study is presumably the first to demonstrate the differential expression of *HSF* genes in plants grown across different regions, which may be related to environmental adaptation and stress.

In abiotic stress, the *HSF* gene family has been reported to significantly respond to stress, including temperature [40], drought [41], and salinity [42], and has the function of helping plants withstand stress [43]. To eliminate differences caused by other factors, such as altitude between different regions, and identify the *CsHSF* gene with the strongest function in heat tolerance, the transcriptome of the hottest TLF region of *Capparis spinosa* was measured for comparison at different temperatures. As a result, eight *CsHSF* genes showed significant differential expression under high-temperature stress, and these genes have been revealed to possess some peculiarities in the previous systematic analysis. For example, *CsHSF06*, *CsHSF12*, *CsHSF19*, and *CsHSF23* have abscisic acid response *cis*-elements on the promoter; *CsHSF05* and *CsHSF12* were upregulated in TLF-specimens and downregulated in YL-specimens, while *CsHSF06*, *CsHSF16*, and *CsHSF23* were upregulated in YL-specimens and downregulated in TLF-specimens. Especially the *CsHSF23* gene, both the *cis*-elements on the promoter and the expression patterns in different regions have shown their response functions to abiotic stress, and its expression changes are the greatest under high temperature. Therefore, it may be the core genes that respond to high temperature in *Capparis spinosa*, and can serve as key genes for further in-depth functional and mechanism research.

## 4. Materials and Methods

### 4.1. The Identification and Chromosome Location of the Capparis spinosa HSF Gene Family

The genome of *Capparis spinosa* was downloaded from the National Genomics Data Center, Beijing Institute of Genomics (accession number GWHBGXB00000000) [26], and was localized for BLAST (version 2.2.31). The mRNAs of known *HSF* genes from all plant species were downloaded from the NCBI nucleotide database and then used as query sequences to search against the *Capparis spinosa* genome [44]. The e-value of Blast was set as 1.0 × 10^−5^ [44]. Meanwhile, the conserved domain of the HSF protein was downloaded from the PFAM website (PF00447) and used as a query to search against the *Capparis spinosa* by HMMER search [44]. The genes obtained from these two methods were then verified by the Conserved Domain Search Tools of NCBI and were renamed as CsHSF01.

The theoretical isoelectric points (pI) and molecular weight of the CsHSF protein were computed by using the ProtParam online tool (https://web.expasy.org/protparam/ (accessed on 16 April 2025)) [45], while their subcellular localization was predicted via the CELLO online tools (http://cello.life.nctu.edu.tw (accessed on 16 April 2025)) [46]. The chromosome location of *CsHSF* genes was retrieved from the caper genome annotation profiles and visualized by the Gene Location Visualization tools of TBtools (version 2.096) [47].

### 4.2. The Gene Structure of the CsHSF Gene Family

The conserved domains and motifs of the *CsHSF* gene family were revealed to show their gene structure. Through the SMART website (http://smart.embl-heidelberg.de (accessed on 30 May 2025)) [48], the DBDs and ODs in the CsHSF family protein sequence were found. Meanwhile, the online tool MEME (https://meme-suite.org/meme/ (accessed on 30 May 2025)) [49] was used to predict the conserved motifs of *HSF* family genes, with the parameter setting to 10 [5]. Combining the conserved domain and motifs contained, the *CsHSF* genes were subdivided into A, B, and C types. The conserved motif and gene structure were visualized by TBtools software.

### 4.3. The Collinearity Analysis of the CsHSF Gene Family

The advanced circus tools in TBtools were used to conduct the collinearity analysis. Firstly, the homologous gene pairs within the caper *HSF* gene family were screened to show the expansion of this family. And then, the whole genome sequences and annotation databases of *Arabidopsis thaliana* (https://www.arabidopsis.org/ (accessed on 17 June 2025)) [50] and *Oryza sativa* (https://riceome.hzau.edu.cn/ (accessed on 17 June 2025)) [51] were taken as references to show the collinearity of *HSF* genes between multiple species.

### 4.4. The Systematic Evolution Analysis of the CsHSF Genes

A total of 24 CsHSF proteins were input to conduct the multiple sequence alignment and phylogenetic tree using MEGA (version 7.0) [52] by the method of Maximum likelihood with a bootstrap of 2000. At the same time, the HSF proteins of *Arabidopsis thaliana* [29] and *Oryza sativa* [53] were taken as the outgroup genes to cluster the evolution and types of family genes. To further evaluate the purification pressure faced by this gene family in evolution, the Ka/Ks ratio (the ratio of non-synonymous substitution rate to synonymous substitution rate) was predicted by a Ka/Ks calculator (V3.0, National Genomics Data Center, Beijing, China) [54].

### 4.5. The Cis-Regulatory Elements of the CsHSF Gene Promoter

The promoter sequences extracted from the upstream 2000 bp of the *CsHSF* gene sequence from the genome of *Capparis spinosa* were analyzed by the online PlantCARE website (https://bioinformatics.psb.ugent.be/webtools/plantcare/html/ (accessed on 21 June 2025)) [55]. The *cis*-regulatory elements obtained in the *CsHSF* gene promoter were statistically analyzed and visualized as a heatmap.

### 4.6. Protein Interaction Network of the CsHSF Genes

To demonstrate the function of *HSF* genes and their interactions with other proteins, the STRING database (https://cn.string-db.org/ (accessed on 13 August 2025)) [56] was used to construct the protein interaction network. All *CsHSF* genes were searched against the STRING database to identify their homologous genes, using *Arabidopsis thaliana* as the reference organism. The nodes of this protein interaction network were then annotated and enriched to show their potential functions and interactions.

### 4.7. The Heatmap and Co-Expression Network of CsHSF Genes

The raw data of *Capparis spinosa* for genome sequencing and assembly were downloaded from the NCBI (https://www.ncbi.nlm.nih.gov/bioproject/PRJNA778809 (accessed on 15 April 2025)), and were transformed to fastq (version 0.12.1) [57]. Quality control was checked using FastQC and then combined by multiQC (version 1.19) [58], followed by sequence alignment against the reference genome using HISAT2 (version 2.2.1) [59]. The expression levels were quantified using the featurecounts (version 2.0.6) [60]. Based on the nine samples of the transcriptome expression database obtained above, the expression of *CsHSF* genes was extracted and visualized as a heatmap using the TBtools software. The co-expression network was then analyzed using the R programming language (version 4.3.3).

### 4.8. The Expression Analysis of CsHSF Genes Under High-Temperature

To show the expression characteristics of *CsHSF* genes at different high temperatures, the leaves of *Capparis spinosa* samples were collected from Turpan, Xinjiang (42°51′10″ N, 88°35′14″ E) in early June (with an average maximum temperature of about 30 °C within three days) and mid-July (with an average maximum temperature of about 40 °C within three days). The *Capparis spinosa* plants at these two time points were in the reproductive stage with no difference in developmental stage. Three samples with similar morphological characteristics (similar age) were selected, and newly developed complete leaves (approximately 4–8 pieces from the stem tip) were collected from their branches.

The cDNA libraries of these samples were constructed and sequenced on the Illumina sequencing platform by Metware Biotechnology Co., Ltd. (Wuhan, China). The clean reads obtained were assembled by Trinity [61], and the integrity of the assembled transcripts was evaluated using the BUSCO software (version 5.4.0) [62]. The clean reads of each sample were aligned with the reference sequence using bowtie2 [63] of the RSEM [64] software, and the number of Mapped Reads and transcript length in the samples were normalized using FPKM. Based on this transcriptome at high temperature, the *CsHSF* genes that were differentially expressed were screened out, and their gene expressions were statistically analyzed as histograms.

## 5. Conclusions

This study identified 24 *HSF* genes from the genome of *Capparis spinosa*, which are distributed on 14 chromosomes. Twenty-four genes belong to three types, and different types of genes contain specific motifs. The family genes have a high degree of collinearity with *Arabidopsis thaliana*, with eight homologous gene pairs within the family, and are undergoing strong purification selection. In gene promoters, *CsHSF* contains 605 *cis*-elements upstream. More than half of the genes showed upregulated expression patterns in different growing regions, with eight genes significantly responding to high-temperature stress. The results can lay the foundation for the study of the function and mechanism of the *HSF* gene family in desert plants. The candidate functional genes obtained can be validated through experiments such as gene cloning and genetic transformation in the future.

## Figures and Tables

**Table 1:** The information on *CsHSF* genes.

Gene Name	Gene Length (bp)	Amino Acids Length (aa)	Number of Exons	Theoretical pI	Molecular Weight	Subcellular Localization
*CsHSF01*	1131	377	2	8.67	42,698.96	Nuclear
*CsHSF02*	609	203	2	8.87	23,996.12	Nuclear
*CsHSF03*	1557	519	3	4.98	57,329.64	Nuclear
*CsHSF04*	936	312	2	8.68	34,004.20	Nuclear/Chloroplast
*CsHSF05*	834	278	2	6.87	30,894.53	Nuclear
*CsHSF06*	903	301	3	5.58	34,702.45	Nuclear
*CsHSF07*	399	133	2	9.16	15,741.72	Nuclear
*CsHSF08*	1161	387	2	5.10	44,645.88	Nuclear
*CsHSF09*	1398	466	4	5.93	52,499.31	Nuclear
*CsHSF10*	972	324	2	5.58	36,742.18	Nuclear
*CsHSF11*	1173	391	2	5.01	44,942.87	Nuclear
*CsHSF12*	852	284	2	7.68	31,455.03	Nuclear
*CsHSF13*	930	310	2	7.11	35,589.58	Nuclear
*CsHSF14*	1008	336	2	9.11	38,324.35	Nuclear
*CsHSF15*	921	307	2	6.35	33,700.77	Nuclear
*CsHSF16*	1095	365	2	5.04	41,020.09	Nuclear
*CsHSF17*	1203	401	4	5.57	45,715.82	Nuclear
*CsHSF18*	1074	358	3	9.22	40,238.60	Nuclear
*CsHSF19*	465	155	2	8.19	16,310.70	Nuclear
*CsHSF20*	1080	360	2	9.22	40,868.05	Nuclear
*CsHSF21*	1482	494	3	5.23	54,416.31	Nuclear
*CsHSF22*	729	243	2	5.41	27,364.39	Nuclear
*CsHSF23*	1032	344	2	5.55	39,703.76	Nuclear
*CsHSF24*	1323	441	2	5.35	49,512.59	Nuclear

**Table 2:** The Ka/Ks ratio between *CsHSF* gene pairs.

Gene Pair		Ka	Ks	Ka/Ks
*CsHSF01*	*CsHSF20*	0.1060	0.4794	0.2210
*CsHSF04*	*CsHSF15*	0.1319	0.4753	0.2775
*CsHSF05*	*CsHSF12*	0.2533	3.0539	0.0829
*CsHSF07*	*CsHSF17*	0.0941	0.5139	0.1831
*CsHSF08*	*CsHSF23*	0.3313	2.3682	0.1399
*CsHSF10*	*CsHSF14*	0.1257	0.3786	0.3321
*CsHSF18*	*CsHSF21*	0.0745	0.2580	0.2889

## Data Availability

The original contributions presented in this study are included in the article. Further inquiries can be directed to the corresponding author.

## Abbreviations

The following abbreviations are used in this manuscript:

KLMY	Karamay
TLF	Turpan
YL	Ili

## Appendix A

**Table A1:** The rename information of *CsHSF* genes.

Gene Name	ID in the Genome	Type
*CsHSF01*	GWHTBGXB001106	B
*CsHSF02*	GWHTBGXB001249	A
*CsHSF03*	GWHTBGXB001406	A
*CsHSF04*	GWHTBGXB002690	B
*CsHSF05*	GWHTBGXB004242	B
*CsHSF06*	GWHTBGXB004942	A
*CsHSF07*	GWHTBGXB005875	A
*CsHSF08*	GWHTBGXB006195	A
*CsHSF09*	GWHTBGXB006266	A
*CsHSF10*	GWHTBGXB006311	C
*CsHSF11*	GWHTBGXB008031	A
*CsHSF12*	GWHTBGXB008188	B
*CsHSF13*	GWHTBGXB012011	B
*CsHSF14*	GWHTBGXB012888	C
*CsHSF15*	GWHTBGXB013272	B
*CsHSF16*	GWHTBGXB014609	A
*CsHSF17*	GWHTBGXB014840	A
*CsHSF18*	GWHTBGXB015614	A
*CsHSF19*	GWHTBGXB017375	A
*CsHSF20*	GWHTBGXB017697	B
*CsHSF21*	GWHTBGXB018704	A
*CsHSF22*	GWHTBGXB019952	B
*CsHSF23*	GWHTBGXB020329	A
*CsHSF24*	GWHTBGXB020674	A

**Table A2:** The genes from out-group species in the phylogenetic tree of the *CsHSF* family.

Species Type	Species	Gene Name	Gene ID
Monocots	*Oryza sativa*	*OsHSFA1*	LOC_Os03g63750
	*Oryza sativa*	*OsHSFA2A*	LOC_Os03g53340
	*Oryza sativa*	*OsHSFA2B*	LOC_Os07g08140
	*Oryza sativa*	*OsHSFA2C*	LOC_Os10g28340
	*Oryza sativa*	*OsHSFA2D*	LOC_Os03g06630
	*Oryza sativa*	*OsHSFA2E*	LOC_Os03g58160
	*Oryza sativa*	*OsHSFA3*	LOC_Os02g32590
	*Oryza sativa*	*OsHSFA4A*	LOC_Os01g54550
	*Oryza sativa*	*OsHSFA4D*	LOC_Os05g45410
	*Oryza sativa*	*OsHSFA5*	LOC_Os02g29340
	*Oryza sativa*	*OsHSFA6*	LOC_Os06g36930
	*Oryza sativa*	*OsHSFA7*	LOC_Os01g39020
	*Oryza sativa*	*OsHSFA9*	LOC_Os03g12370
	*Oryza sativa*	*OsHSFB1*	LOC_Os09g28354
	*Oryza sativa*	*OsHSFB2A*	LOC_Os04g48030
	*Oryza sativa*	*OsHSFB2B*	LOC_Os08g43334
	*Oryza sativa*	*OsHSFB2C*	LOC_Os09g35790
	*Oryza sativa*	*OsHSFB4A*	LOC_Os08g36700
	*Oryza sativa*	*OsHSFB4B*	LOC_Os07g44690
	*Oryza sativa*	*OsHSFB4C*	LOC_Os09g28200
	*Oryza sativa*	*OsHSFB4D*	LOC_Os03g25120
	*Oryza sativa*	*OsHSFC1A*	LOC_Os01g43590
	*Oryza sativa*	*OsHSFC1B*	LOC_Os01g53220
	*Oryza sativa*	*OsHSFC2A*	LOC_Os02g13800
	*Oryza sativa*	*OsHSFC2B*	LOC_Os06g35960
Eudicots	*Arabidopsis thaliana*	*AtHSFA1A*	AT4G17750.1
	*Arabidopsis thaliana*	*AtHSFA1B*	AT5G16820.1
	*Arabidopsis thaliana*	*AtHSFA1D*	AT1G32330
	*Arabidopsis thaliana*	*AtHSFA1E*	AT3G02990
	*Arabidopsis thaliana*	*AtHSFA2*	AT2G26150
	*Arabidopsis thaliana*	*AtHSFA3*	AT5G03720
	*Arabidopsis thaliana*	*AtHSFA4A*	AT4G18880
	*Arabidopsis thaliana*	*AtHSFA4C*	AT5G45710
	*Arabidopsis thaliana*	*AtHSFA5*	AT4G13980
	*Arabidopsis thaliana*	*AtHSFA6A*	AT5G43840
	*Arabidopsis thaliana*	*AtHSFA6B*	AT3G22830.1
	*Arabidopsis thaliana*	*AtHSFA7A*	AT3G51910.1
	*Arabidopsis thaliana*	*AtHSFA7B*	AT3G63350.1
	*Arabidopsis thaliana*	*AtHSFA8*	AT1G67970.1
	*Arabidopsis thaliana*	*AtHSFA9*	AT5G54070.1
	*Arabidopsis thaliana*	*AtHSFB1*	AT4G36990.1
	*Arabidopsis thaliana*	*AtHSFB2A*	AT5G62020.1
	*Arabidopsis thaliana*	*AtHSFB2B*	AT4G11660.1
	*Arabidopsis thaliana*	*AtHSFB3*	AT2G41690.1
	*Arabidopsis thaliana*	*AtHSFB4*	AT1G46264.1
	*Arabidopsis thaliana*	*AtHSFC1*	AT3G24520.1

## References

[1] Breshears D.D., Fontaine J.B., Ruthrof K.X., Field J.P., Feng X., Burger J.R., Law D.J., Kala J., Hardy G.E.S.J. (2021). Underappreciated plant vulnerabilities to heat waves. New Phytol..

[2] Méndez-Vallejo C., Simpson N., Johnson F., Birt A. (2023). Climate Change 2023: Synthesis Report (Full Volume) Contribution of Working Groups I, II and III to the Sixth Assessment Report of the Intergovernmental Panel on Climate Change.

[3] Andrási N., Pettkó-Szandtner A., Szabados L. (2021). Diversity of plant heat shock factors: Regulation, interactions, and functions. J. Exp. Bot..

[4] Wang X., Shi X., Chen S., Ma C., Xu S. (2018). Evolutionary Origin, Gradual Accumulation and Functional Divergence of Heat Shock Factor Gene Family with Plant Evolution. Front. Plant Sci..

[5] Yu T., Bai Y., Liu Z., Wang Z., Yang Q., Wu T., Feng S., Zhang Y., Shen S., Li Q. (2022). Large-scale analyses of heat shock transcription factors and database construction based on whole-genome genes in horticultural and representative plants. Hortic. Res..

[6] Goel K., Kundu P., Gahlaut V., Sharma P., Kumar A., Thakur S., Verma V., Bhargava B., Chandora R., Zinta G. (2023). Functional divergence of Heat Shock Factors (Hsfs) during heat stress and recovery at the tissue and developmental scales in C4 grain amaranth (*Amaranthus hypochondriacus*). Front. Plant Sci..

[7] Xue G.-P., Sadat S., Drenth J., McIntyre C.L. (2013). The heat shock factor family from *Triticum aestivum* in response to heat and other major abiotic stresses and their role in regulation of heat shock protein genes. J. Exp. Bot..

[8] Agarwal P., Khurana P. (2019). Functional characterization of HSFs from wheat in response to heat and other abiotic stress conditions. Funct. Integr. Genom..

[9] Duan S., Liu B., Zhang Y., Li G., Guo X. (2019). Genome-wide identification and abiotic stress-responsive pattern of heat shock transcription factor family in *Triticum aestivum* L. BMC Genom..

[10] Yun L., Zhang Y., Li S., Yang J., Wang C., Zheng L., Ji L., Yang J., Song L., Shi Y. (2023). Phylogenetic and expression analyses of HSF gene families in wheat (*Triticum aestivum* L.) and characterization of TaHSFB4-2B under abiotic stress. Front. Plant Sci..

[11] Yabuta Y. (2016). Functions of heat shock transcription factors involved in response to photooxidative stresses in Arabidopsis. Biosci. Biotechnol. Biochem..

[12] Gomez-Pastor R., Burchfiel E.T., Thiele D.J. (2017). Regulation of heat shock transcription factors and their roles in physiology and disease. Nat. Rev. Mol. Cell Biol..

[13] Ikeda M., Mitsuda N., Ohme-Takagi M. (2011). Arabidopsis HsfB1 and HsfB2b act as repressors of the expression of heat-inducible Hsfs but positively regulate the acquired thermotolerance. Plant Physiol..

[14] Peng S., Zhu Z., Zhao K., Shi J., Yang Y., He M., Wang Y. (2013). A Novel Heat Shock Transcription Factor, VpHsf1, from Chinese Wild Vitis pseudoreticulata is Involved in Biotic and Abiotic Stresses. Plant Mol. Biol. Report..

[15] Röth S., Mirus O., Bublak D., Scharf K.-D., Schleiff E. (2016). DNA-binding and repressor function are prerequisites for the turnover of the tomato heat stress transcription factor HsfB1. Plant J..

[16] Liang Y., Wang J., Zheng J., Gong Z., Li Z., Ai X., Li X., Chen Q. (2021). Genome-Wide Comparative Analysis of Heat Shock Transcription Factors Provides Novel Insights for Evolutionary History and Expression Characterization in Cotton Diploid and Tetraploid Genomes. Front. Genet..

[17] Ceylan Y., Altunoglu Y.C., Horuz E. (2023). HSF and Hsp Gene Families in sunflower: A comprehensive genome-wide determination survey and expression patterns under abiotic stress conditions. Protoplasma.

[18] Zhao S., Qing J., Yang Z., Tian T., Yan Y., Li H., Bai Y.E. (2024). Genome-Wide Identification and Expression Analysis of the HSF Gene Family in *Ammopiptanthus mongolicus*. Curr. Issues Mol. Biol..

[19] Wang M., Yuan X., Xu L. (2023). Germplasm characterization and SDS-PAGE analysis of caper (*Capparis spinosa* L.) from different provenances. BMC Plant Biol..

[20] Kdimy A., El Yadini M., Guaadaoui A., Bourais I., El Hajjaji S., Le H.V. (2022). Phytochemistry, Biological Activities, Therapeutic Potential, and Socio-Economic Value of the Caper Bush (*Capparis Spinosa* L.). Chem. Biodivers..

[21] Chedraoui S., Abi-Rizk A., El-Beyrouthy M., Chalak L., Ouaini N., Rajjou L. (2017). *Capparis spinosa* L. in A Systematic Review: A Xerophilous Species of Multi Values and Promising Potentialities for Agrosystems under the Threat of Global Warming. Front. Plant Sci..

[22] Annaz H., Sane Y., Bitchagno G.T.M., Ben Bakrim W., Drissi B., Mahdi I., El Bouhssini M., Sobeh M. (2022). Caper (*Capparis spinosa* L.): An Updated Review on Its Phytochemistry, Nutritional Value, Traditional Uses, and Therapeutic Potential. Front. Pharmacol..

[23] Rhizopoulou S., Psaras G.K. (2003). Development and structure of drought-tolerant leaves of the Mediterranean shrub *Capparis spinosa* L. Ann. Bot..

[24] Afzali S.F., Sadeghi H., Taban A. (2023). A comprehensive model for predicting the development of defense system of *Capparis spinosa* L.: A novel approach to assess the physiological indices. Sci. Rep..

[25] Ashraf U., Chaudhry M.N., Ahmad S.R., Ashraf I., Arslan M., Noor H., Jabbar M. (2018). Impacts of climate change on *Capparis spinosa* L. based on ecological niche modeling. PeerJ.

[26] Wang L., Fan L., Zhao Z., Zhang Z., Jiang L., Chai M., Tian C. (2022). The *Capparis spinosa* var. herbacea genome provides the first genomic instrument for a diversity and evolution study of the Capparaceae family. Gigascience.

[27] Zhu S., Wang H., Xue Q., Zou H., Liu W., Xue Q., Ding X.-Y. (2023). Genome-wide identification and expression analysis of growth-regulating factors in *Dendrobium officinale* and *Dendrobium chrysotoxum*. PeerJ.

[28] Huang L., Zhang L., Zhang P., Liu J., Li L., Li H., Wang X., Bai Y., Jiang G., Qin P. (2024). Molecular characteristics and expression pattern of the FAR1 gene during spike sprouting in quinoa. Sci. Rep..

[29] Wang X., Zhu Y., Tang L., Wang Y., Sun R., Deng X. (2024). Arabidopsis HSFA9 Acts as a Regulator of Heat Response Gene Expression and the Acquisition of Thermotolerance and Seed Longevity. Plant Cell Physiol..

[30] Zhang Y., Wang C., Wang C., Yun L., Song L., Idrees M., Liu H., Zhang Q., Yang J., Zheng X. (2022). OsHsfB4b Confers Enhanced Drought Tolerance in Transgenic Arabidopsis and Rice. Int. J. Mol. Sci..

[31] Yang X., Wang S., Cai J., Zhang T., Yuan D., Li Y. (2024). Genome-wide identification, phylogeny and expression analysis of Hsf gene family in *Verbena bonariensis* under low-temperature stress. BMC Genom..

[32] Mishra S.K., Tripp J., Winkelhaus S., Tschiersch B., Theres K., Nover L., Scharf K.-D. (2002). In the complex family of heat stress transcription factors, HsfA1 has a unique role as master regulator of thermotolerance in tomato. Genes Dev..

[33] Li X.-D., Wang X.-L., Cai Y.-M., Wu J.-H., Mo B.-T., Yu E.-R. (2017). Arabidopsis heat stress transcription factors A2 (HSFA2) and A3 (HSFA3) function in the same heat regulation pathway. Acta Physiol. Plant..

[34] Hahn A., Bublak D., Schleiff E., Scharf K.-D. (2011). Crosstalk between Hsp90 and Hsp70 chaperones and heat stress transcription factors in tomato. Plant Cell.

[35] Wu T.-Y., Hoh K.L., Boonyaves K., Krishnamoorthi S., Urano D. (2022). Diversification of heat shock transcription factors expanded thermal stress responses during early plant evolution. Plant Cell.

[36] Rehman A., Atif R.M., Azhar M.T., Peng Z., Li H., Qin G., Jia Y., Pan Z., He S., Qayyum A. (2021). Genome wide identification, classification and functional characterization of heat shock transcription factors in cultivated and ancestral cottons (*Gossypium* spp.). Int. J. Biol. Macromol..

[37] Zang D., Wang J., Zhang X., Liu Z., Wang Y. (2019). Arabidopsis heat shock transcription factor HSFA7b positively mediates salt stress tolerance by binding to an E-box-like motif to regulate gene expression. J. Exp. Bot..

[38] Liu J., Sun N., Liu M., Liu J., Du B., Wang X., Qi X. (2013). An autoregulatory loop controlling Arabidopsis HsfA2 expression: Role of heat shock-induced alternative splicing. Plant Physiol..

[39] Li H.-G., Yang L., Fang Y., Wang G., Lyu S., Deng S. (2024). A genome-wide-level insight into the HSF gene family of *Rhodomyrtus tomentosa* and the functional divergence of RtHSFA2a and RtHSFA2b in thermal adaptation. Plant Physiol. Biochem..

[40] Mishra S.K., Chaudhary C., Baliyan S., Poonia A.K., Sirohi P., Kanwar M., Gazal S., Kumari A., Sircar D., Germain H. (2024). Heat-stress-responsive HvHSFA2e gene regulates the heat and drought tolerance in barley through modulation of phytohormone and secondary metabolic pathways. Plant Cell Rep..

[41] Kanwar M., Chaudhary C., Anand K.A., Singh S., Garg M., Mishra S.K., Sirohi P., Chauhan H. (2023). An insight into *Pisum sativum* HSF gene family-Genome-wide identification, phylogenetic, expression, and analysis of transactivation potential of pea heat shock transcription factor. Plant Physiol. Biochem..

[42] Yuan T., Liang J., Dai J., Zhou X.-R., Liao W., Guo M., Aslam M., Li S., Cao G., Cao S. (2022). Genome-Wide Identification of Eucalyptus Heat Shock Transcription Factor Family and Their Transcriptional Analysis under Salt and Temperature Stresses. Int. J. Mol. Sci..

[43] Fragkostefanakis S., Schleiff E., Scharf K.-D. (2025). Back to the basics: The molecular blueprint of plant heat stress transcription factors. Biol. Chem..

[44] Li L., Lv B., Zang K., Jiang Y., Wang C., Wang Y., Wang K., Zhao M., Chen P., Lei J. (2023). Genome-wide identification and systematic analysis of the HD-Zip gene family and its roles in response to pH in *Panax ginseng* Meyer. BMC Plant Biol..

[45] Wilkins M.R., Gasteiger E., Bairoch A., Sanchez J.C., Williams K.L., Appel R.D., Hochstrasser D.F. (1999). Protein identification and analysis tools in the ExPASy server. Methods Mol. Biol..

[46] Yu C.-S., Lin C.-J., Hwang J.-K. (2004). Predicting subcellular localization of proteins for Gram-negative bacteria by support vector machines based on n-peptide compositions. Protein Sci..

[47] Chen C., Chen H., Zhang Y., Thomas H.R., Frank M.H., He Y., Xia R. (2020). TBtools: An Integrative Toolkit Developed for Interactive Analyses of Big Biological Data. Mol. Plant.

[48] Letunic I., Bork P. (2025). SMART v10: Three decades of the protein domain annotation resource. Nucleic Acids Res..

[49] Bailey T.L., Boden M., Buske F.A., Frith M., Grant C.E., Clementi L., Ren J., Li W.W., Noble W.S. (2009). MEME SUITE: Tools for motif discovery and searching. Nucleic Acids Res..

[50] Swarbreck D., Wilks C., Lamesch P., Berardini T.Z., Garcia-Hernandez M., Foerster H., Li D., Meyer T., Muller R., Ploetz L. (2007). The Arabidopsis Information Resource (TAIR): Gene structure and function annotation. Nucleic Acids Res..

[51] Yu Z., Chen Y., Zhou Y., Zhang Y., Li M., Ouyang Y., Chebotarov D., Mauleon R., Zhao H., Xie W. (2023). Rice Gene Index: A comprehensive pan-genome database for comparative and functional genomics of Asian rice. Mol. Plant.

[52] Kumar S., Stecher G., Li M., Knyaz C., Tamura K. (2018). MEGA X: Molecular Evolutionary Genetics Analysis across Computing Platforms. Mol. Biol. Evol..

[53] Shamshad A., Rashid M., Zaman Q.U. (2023). In-silico analysis of heat shock transcription factor (OsHSF) gene family in rice (*Oryza sativa* L.). BMC Plant Biol.

[54] Zhang Z. (2022). KaKs_Calculator 3.0: Calculating Selective Pressure on Coding and Non-coding Sequences. Genom. Proteom. Bioinform..

[55] Zhu L., Huang C., Yuan C., Liu Y., Yu H., Long Y., Zeng J. (2025). Genome-wide identification and characterization of NBS-LRR gene family in tobacco (*Nicotiana benthamiana*). Sci. Rep..

[56] Szklarczyk D., Kirsch R., Koutrouli M., Nastou K., Mehryary F., Hachilif R., Gable A.L., Fang T., Doncheva N.T., Pyysalo S. (2023). The STRING database in 2023: Protein-protein association networks and functional enrichment analyses for any sequenced genome of interest. Nucleic Acids Res..

[57] Wingett S.W., Andrews S. (2018). FastQ Screen: A tool for multi-genome mapping and quality control. F1000Res.

[58] Ewels P., Magnusson M., Lundin S., Käller M. (2016). MultiQC: Summarize analysis results for multiple tools and samples in a single report. Bioinformatics.

[59] Kim D., Paggi J.M., Park C., Bennett C., Salzberg S.L. (2019). Graph-based genome alignment and genotyping with HISAT2 and HISAT-genotype. Nat. Biotechnol..

[60] Liao Y., Smyth G.K., Shi W. (2013). featureCounts: An efficient general purpose program for assigning sequence reads to genomic features. Bioinformatics.

[61] Grabherr M.G., Haas B.J., Yassour M., Levin J.Z., Thompson D.A., Amit I., Adiconis X., Fan L., Raychowdhury R., Zeng Q. (2011). Full-length transcriptome assembly from RNA-Seq data without a reference genome. Nat. Biotechnol..

[62] Simão F.A., Waterhouse R.M., Ioannidis P., Kriventseva E.V., Zdobnov E.M. (2015). BUSCO: Assessing genome assembly and annotation completeness with single-copy orthologs. Bioinformatics.

[63] Langmead B., Salzberg S.L. (2012). Fast gapped-read alignment with Bowtie 2. Nat. Methods.

[64] Li B., Dewey C.N. (2011). RSEM: Accurate transcript quantification from RNA-Seq data with or without a reference genome. BMC Bioinform..

